# Bioinks and biofabrication techniques for biosensors development: A review

**DOI:** 10.1016/j.mtbio.2024.101185

**Published:** 2024-08-05

**Authors:** Róisín Byrne, Amanda Carrico, Mariagrazia Lettieri, Athira K. Rajan, Robert J. Forster, Loanda R. Cumba

**Affiliations:** a School of Chemical Sciences, National Centre for Sensor Research, Dublin City University, Glasnevin, Dublin 9, Ireland; b FutureNeuro, The SFI Research Centre for Chronic and Rare Neurological Diseases, Royal College of Surgeons, Ireland

**Keywords:** Advanced bioinks, Bioink-based biosensors, Biofabrication, Bioprinting technologies

## Abstract

3D bioprinting technologies and bioink development are enabling significant advances in miniaturized and integrated biosensors. For example, bioreceptors can be immobilized within a porous 3D structure to significantly amplify the signal, while biocompatible and mechanically flexible systems uniquely enable wearable chem- and bio-sensors. This advancement is accelerating translation by enabling the production of high performance, reproducible, and flexible analytical devices. The formulation of the bioink plays a crucial role in determining the bio-functionality of the resulting printed structures, e.g., the porosity that allows the analyte to diffuse through the 3D structure, the affinity and avidity of the receptors, etc. This review explores the next generation of advanced bioinks for biosensor development and provides insights into the latest cutting-edge bioprinting technologies. The bioprinting methods available for biosensor fabrication including inkjet, extrusion, and laser-based bioprinting, are discussed. The advantages and limitations of each method are analysed, and recent advancements in bioprinting technologies are presented. The review then delves into the properties of advanced bioinks, such as biocompatibility, printability, stability, and applicability. Different types of advanced bioinks are explored, including multicomponent, stimuli-responsive, and conductive bioinks. Finally, the next generation of bioinks for biosensors is considered, identifying possible new opportunities and challenges. Overall, this literature review highlights the combined importance of bioink formulation and bioprinting methods for the development of high-performance analytical biosensors.

## Graphical abstract

**Figure:**
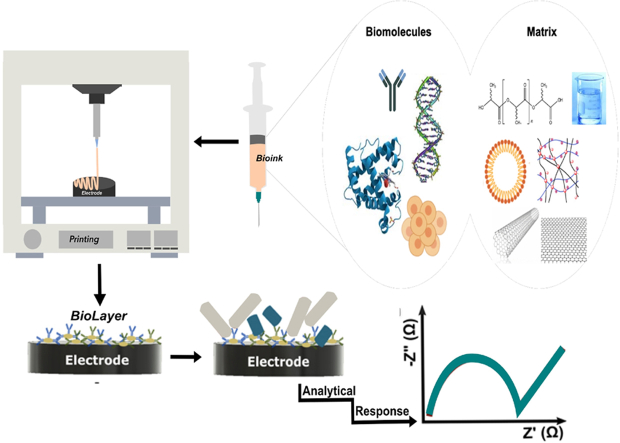

## 1 Introduction

Recently, the development of novel bioinks with tailored physical-chemical properties has emerged as an important approach for the development of high performance (bio)sensors. These sensors have distinctive properties including wide dynamic ranges, low limits of detection, high analytical sensitivity as well as mechanical flexibility and biocompatibility that open new opportunities such as implantable or wearable sensors. This combination of features can address significant contemporary challenges, such as the rapid detection of biofilms in built environments, the enhancement of medical devices and food safety, the identification of environmental contaminants, and the low-cost, rapid, and sensitive detection of disease biomarkers. Moreover, the novel bioinks can also advance the development of multifunctional tissues, organs, disease modelling, and personalized and regenerative medicine, as well as cosmetic and aesthetic applications [[1], [2], [3], [4]].

Bioinks are typically composed of biocompatible materials that seek to mimic the properties of the extracellular matrix (ECM) providing an environment that is conducive to preserving the functionality of biomolecules, e.g., biomolecule binding reactions, as well as cell growth and development [5]. However, recent scientific progress in bioink formulations includes the incorporation of different types of biomolecules, e.g.*,* antibodies, enzymes, nucleic acids [6] *and* advanced functional materials, e.g., graphene, metal nanoparticles, carbon nanotubes and stimuli-responsive polymers, to enhance mechanical strength, optical properties and electrical conductivity [7].

An emerging field is the application of multifunctional advanced bioinks in the biosensor development area. Biosensors have gained significant attention in recent years due to their applications in medical diagnostics [8], environmental monitoring [9], food safety [10], and many other fields [11]. Among these, electrochemical biosensors stand out as analytical devices that integrate biological elements, such as enzymes, antibodies, or cells, with a transducer to detect and quantify specific analytes [12]. Of particular note is glucose monitoring, where more than 4 billion tests are sold annually worldwide testifying to the significant competitive advantages of electrochemical detection. However, to democratize biosensors, many challenges exist, including the issue of the (perceived) pain of collecting a finger prick blood sample which drives interest in minimally invasive samples such as saliva, tears, perspiration, or urine. Achieving these goals demands the design and fabrication of biocompatible materials that can effectively interface with biological systems but are also electrically conducting which could lead to implantable, wearable, and analytical, sample-to-answer systems. Moreover, to address the growing cost of healthcare globally, biomarker measurement must move out of the centralized testing laboratory and be closer to the patient. Bioinks are promising candidates for the development of point-of-care (POC) biosensors, due to their ability to create complex 3D structures that mimic the natural environment that is conducive to the optimum performance of the bioreceptor. Bioprinting enables the precise deposition of bioinks in a layer-by-layer manner, facilitating the creation of complex 3D architectures with high resolution and reproducibility [13]. Such precision is essential for the accurate placement of biological elements within the transducer leading to high performance biosensors [14]. This level of customization allows the development of unique bioinks, enabling the creation of highly specific and sensitive biosensors, thereby improving accuracy and reliability. Bioprinting processes can also provide solutions to effectively replace the need for animal testing [15].

This review focuses on recent advancements in bioprinting methods and bioink formulations for the development of biosensors. Specifically, it assesses the latest research and innovations of advanced bioinks and bioprinting methods, alongside discussing the current obstacles and future directions in the development of bioink-based biosensors, highlighting the potential for these technologies to revolutionize the manufacturing process of POC devices.

## 2 Bioprinting methods

Bioprinting technologies can create engineered complex structures containing active biomolecules through precise positioning and sequential layer-by-layer deposition of biologically relevant materials [16,17].

Advanced bioinks are materials that contain encapsulated active biomolecules in a non-cytotoxic matrix, comprised of natural and synthetic polymers, additives, and solvents. Therefore, they are optimally used in bioprinting as they are designed to improve printability, bioactivity, biocompatibility and crosslinking of the printed structures outside the “biofabrication window” [18,19]. The biofabrication window represents the optimal set of conditions for successfully bioprinting active biomolecules and creating functional biological structures [20]. Otherwise, the printed structure can become compromised, e.g., by chemical cytotoxicity caused by the core matrix or pressure-induced bio-inactivation effect during the biomaterial extrusion [21].

**Fig. 1:**
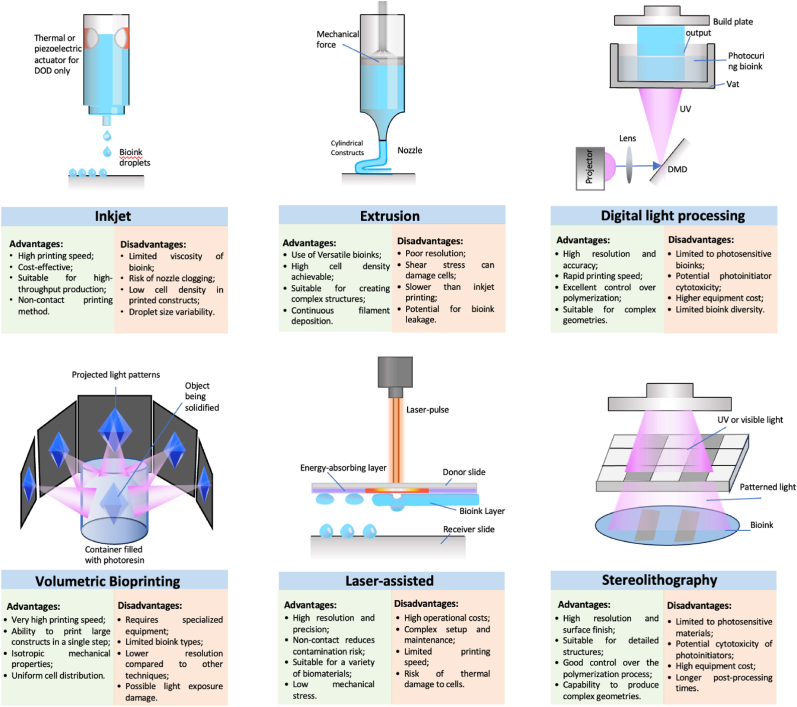
Advantage and disadvantages of biofabrication technologies for biosensors development.

Prior to bioink development, it is crucial to assess which bioprinting method will best reproduce the physiochemical, architectural, and mechanical complexity required by the sensor, the tissue or biostructure. These methods include inkjet [22], extrusion-based [23], light-based [24], volumetric [25], laser-assisted [26], and stereolithography [27]. Here, the challenges, applications, and prospects of the different bioprinting methods are discussed. Fig. 1 presents the advantages and disadvantages of each biofabrication process discussed in this review.

### 2.1 Inkjet-based bioprinting

In 2003, Bolan and co-workers developed one of the first inkjet-bioprinting approaches [28]. The group modified a standard inkjet printer to dispense a suspension of cells and proteins that were repeatedly printed on a layer of poly[N-isopropylacrylamide-*co*-2-(N,N-dimethylamino)ethyl acrylate] on a collagen substrate, enhancing the construct thickness [29]. This experiment paved the way for modern inkjet printing, now recognized as a reprographic method, for the controlled and precise deposition of small drops of ink onto a substrate. It can be broken down into three stages: *(1)* the forced release of the ink in the form of droplets, *(2)* solid-liquid interaction, after droplet positioning onto the substrate and *(3)* the drying and solidifying of droplets to form printed structures [30].

*Continuous inkjet printing* (CIJ) and *drop-on-demand printing* (DOD) are two subcategories of inkjet printing that explain the two types of physical processes that can occur upon droplet generation used in bioprinting [31]. As the name suggests, continuous inkjet printers produce a continuous flow of ink where the droplets generated are typically twice the size of the nozzle opening. The material is dispensed from the nozzle of 50–80 μm induced by a high-pressure pump under an electric field. Eventually, these droplets coalesce into a single stream of material [32]. Usually, DOD printing involves multiple nozzles, where a voltage is applied to a piezoelectric actuator causing a sudden change in material volume resulting in a pressure wave that extrudes the ink throughout the capillary. When the kinetic energy transferred outwards is larger than the surface energy needed to form a droplet, the ink is ejected. The velocity of the droplet also depends on this energy transfer [33]. Unlike CIJ printing, the volume of droplets formed with DOD printing is in accordance with the nozzle diameter. Moreover, CIJ recirculates the bioink, so DOD is favoured to avoid contamination [34] being more suitable for the fabrication of biosensors [35], electronic devices [36], and cell-based assay systems [37].

**Fig. 2:**
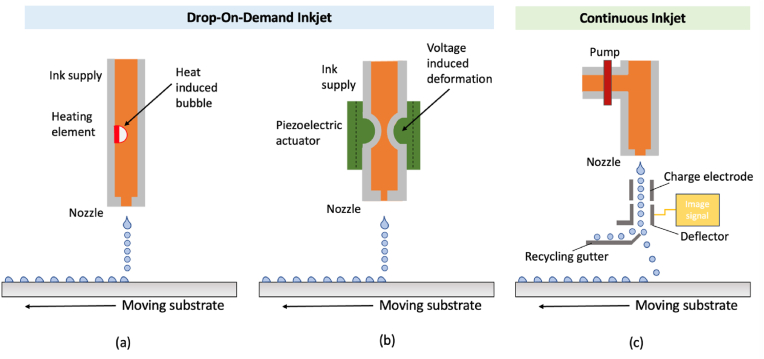
Schematic representation of drop-on-demand inkjet bioprinting, (a) thermal inkjet printing, (b) piezoelectric printing and (c) continuous inkjet bioprinting adapted from Ref. [40]. Licensee MDPI, Basel, Switzerland (CC-BY 4.0).

To summarize, the mechanism of inkjet printing involves the generation and subsequent loss of microbubbles within the nozzle causing a build-up of pressure, which ejects droplets of material from the cartridge. These microbubbles are most commonly generated from *thermal* [38] or *piezoelectric* [39] effects (Fig. 2).

*Thermal inkjet printing* is a very commonly used noncontact DOD printing system due to its reproducibility, low cost, and high throughput [41]. The thermal actuator heats the bioink to generate a swell of vapor, which generates a pressure pulse essential for droplet emission through the nozzle. The chamber is refilled when the current is withdrawn, which consequently reduces the temperature and pressure creating a vacuum [42]. The prime concern with thermal-based printers is the increased risk of thermal stress, the denaturation, and deactivation of the biomolecules in the bioink formulation. Bolan and collaborators stated a 15 % decrease in enzyme activity after thermal bioprinting [43]. Campbell et al. observed an apoptotic rate for post-bioprinted MCF7 breast cancer cells of 69 % after 24 h [44]. Furthermore, stability, directionality, uneven droplet size, and difficulty achieving droplets at submicron level have also been reported [45]. Therefore, piezoelectric printers are more often used to print bioinks as the nozzle temperature is easily controlled and solvents with low boiling points can be used, unlike thermal inkjet printing [46].

*Piezoelectric inkjet printing* presents a solution to the limitations posed by thermal inkjet methods. This established and reliable technique employs a transducer to manipulate acoustic waves for bioink ejection. In essence, an electric pulse is applied, the plate distorts producing a pressure wave that ejects the ink, returning the transducer to its original state once the pulse subsides. Due to dampening of the pressure waves, highly concentrated and viscous bioinks are not recommended as it will hinder ink ejection [41].

AlChamaa et al. used a piezoelectric DMP-2850 Fujifilm Dimatix printer to bioprint a highly sensitive point-of-care biosensor by ‘re-inventing’ organic electrochemical transistors [42]. Bovine serum albumin (BSA) proteins were detected with a physiologically relevant limit of detection of 1 pM. The study demonstrates a scalable, reproducible, and low-cost fabrication process with the potential of inkjet printing biomarkers for cardiac diseases in an accurate and reliable way. However, the limitation of inkjet printing (*i.e.* biomolecules stability) needs to be considered to a greater extent to progress in the biosensing area [47].

**Fig. 3:**
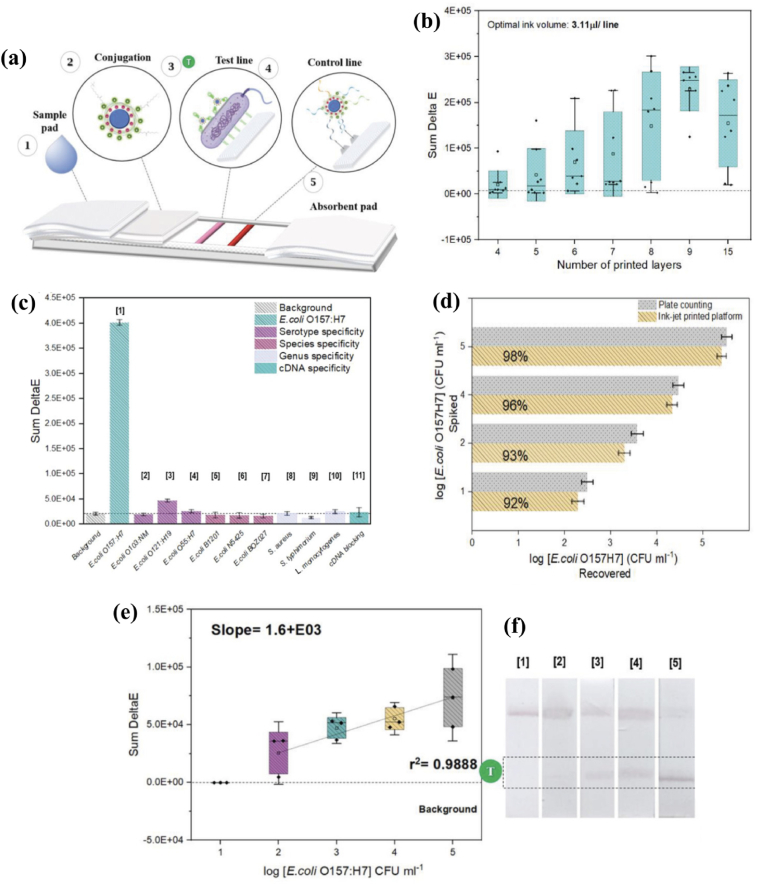
Novel paper-based chromatographic biosensor platform: (a) graphical illustration of the aptamer-based detection mechanism, (b) optimization of the number of printed layers based on the colour response; (c) Specificity test - All interferent bacteria were tested at a high concentration (10^6^ CFU mL^−1^); (d) Recovery test for the proposed platform and validation of the proposed approach compared with the conventional plate counting technique; (e) Correlation plot: grey intensity (Sum DeltaE) versus Cell concentration (*E. coli*); (f) Selection of real images acquired by an EPSON1000XL flatbed scanner. The figure was adapted and reprinted from Ref. [48] with the permission of WILEY-VCH Verlag GmbH & Co. KGaA, Weinheim. (For interpretation of the references to color in this figure legend, the reader is referred to the Web version of this article.)

Díaz-Amaya and co-workers developed a piezoelectric inkjet-bioprinted novel paper-based chromatographic platform containing DNA-based biocapture molecules on a nitrocellulose substrate. Multilayers of the bioink were deposited and tested, where nine printed layers provide an optimal signal response and improve the variability ratio among the independent measurements (Fig. 3). After the bioprinting process, the biosensor presented high specificity against 10 interferent bacteria strains, good sensitivity (LOD 233 CFU mL^−1^ in ground beef), and extremely stable over time storage (10 weeks) at room temperature [48].

To date, inkjet-based bioprinting is considered a robust approach for its cost-effectiveness, high-throughput, accuracy, repeatability, and high resolution (up to 50 μm) [22,43,49]. The multiple nozzles feature offers the advantage of printing different bioinks that contains distinct active biomolecules enabling the development of complex tissue models [50], pathogenic biofilms [51] and biosensors [52]. However, the use of low viscosity bioinks in the inkjet bioprinting process is unavoidable as it may compromise the stability and resolution of the constructs due to the uneven formation of droplets and deactivation of biomolecules induced by thermal or mechanical stress [53].

### 2.2 Extrusion-based bioprinting

Extrusion-based bioprinting is the most popular biofabrication method renowned for its simplicity, cost-effectiveness, and scalability. Unlike inkjet bioprinting, it is particularly suitable for printing with bioinks over a wide range of viscosities [54]. The process involves the continuous ejection of the bioink through a nozzle or needle by pneumatic pressure, piston, or a screw mechanism to create a 3D structure [55]. Due to considerations regarding shape fidelity, the possible adverse effects on cell viability, and the element of shear thinning, the bioprintable materials need to be carefully selected and formulated.

For example, low viscosity bioinks, may result in poor printability and low resolution, while high viscosity bioinks can improve printability and shape fidelity, but may require increased extrusion pressure, potentially causing biomolecule inactivation or cell damage/death [56]. Shear-thinning bioinks are the most suitable for extrusion-based bioprinting processes due to its viscosity-recovery behaviour after printing, i.e., the viscosity change when subjected to a deforming force [57].

Despite these challenges, extrusion-based 3D-bioprinting shows promise, with studies demonstrating its capability to print human-scale tissue, a feature yet to be accomplished by other bioprinting methods [58]. Moreover, it offers significant potential for innovation in the biosensor manufacturing due to its ease to use, high printability, and resolution.

Among the bioprinting technologies, *fused deposition modelling* (FDM) is widely employed for constructing complex structures at low cost [59]. FDM relies on the extrusion of molten polymers through a high-temperature nozzle to build 3D constructs in a layer-by-layer sequence. The melted material is extruded, cooled, and solidified, creating a fused 3D structure. Thermoplastic materials like acrylonitrile butadiene styrene, polycarbonate, and polylactic acid are well-suited for FDM due to their low melting temperatures, low-cost, and processability. Actuators control the 3D structural formation in x, y, and z dimensions by regulating the nozzle orientation in such a fashion that new layers are built on top of the previous ones. The movement and speed of the nozzle can be easily altered, thus controlling the width and thickness of the layer deposition [60].

Although FDM is suitable for the rapid design and testing of prototypes in medical devices and sensor housings, the high temperature used during the extrusion step can negatively affect cell viability, as well as induce biomolecule inactivation and denaturation. This limitation can be addressed by using the *direct ink writing* (DIW) technique, which can be performed at room temperature.

Balasubramanian et al. reported a simple approach for spatial patterning of different *E. coli* strains onto agar substrates employing a customized do-it-yourself 3D printer. They studied the biological endurance of bacterial biofilms by tuning of the bioink composition to alter bacterial, or cellulose density [61].

**Fig. 4:**
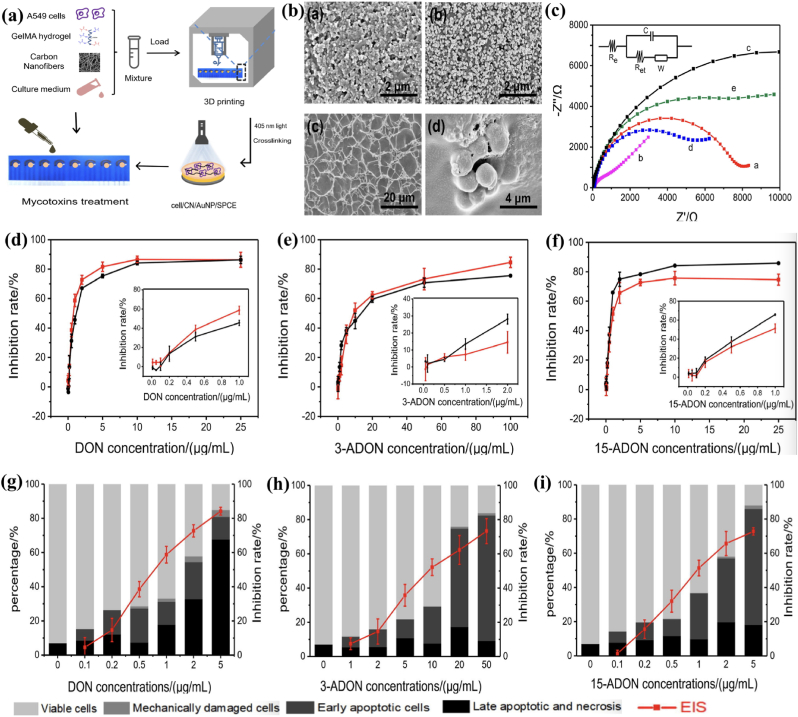
(a) Schematic representation of the route for the preparation of the 3D printed electrochemical cell-based biosensor; (b) SEM images of the main steps in electrode fabrication: a. bare SPCE electrode, b. AuNP/SPCE-modified electrode, c. CN/GelMA/AuNP/SPCE-modified electrode, d. Cluster cells grown in CN/GelMA composite hydrogels; (c) Nyquist diagrams of electrochemical impedance spectra of a. bare SPCE, b. AuNP/SPCE, c. GelMA/AuNP/SPCE, d. CN/GelMA/AuNP/SPCE, e. cell/CN/GelMA/AuNP/SPCE. The insert is the equivalent circuit model, to which all of the experimental impedance curves were fitted; (d–f) Cytotoxicity curves for the A549 cells exposed to DON, 3-ADON, and 15-ADON for 48 h, as determined using the CCK-8 assay (black line) and the proposed electrochemical method (red line); (g-i)Effects of DON and its acetylated derivatives on A549 cell viability using the proposed electrochemical method and biological assay EIS and cell apoptosis. The figure was adapted and reprinted from Ref. [62] with permission of Elsevier. (For interpretation of the references to color in this figure legend, the reader is referred to the Web version of this article.)

To non-invasively monitor the viability of 3D printed cells and evaluate the individual or combined toxicity of deoxynivalenol (DON), 3-acetyldeoxynivalenol (3-ADON), and 15-acetyldeoxynivalenol (15-ADON), K. Wei and collaborators [62], developed a “honeycomb”/screen-printed electrode based electrochemical biosensor (Fig. 4). Mycotoxin toxicity was monitored using electrochemical impedance spectroscopy (EIS), and results indicated that DON, 3-ADON, and 15-ADON caused significant decreases in cell viability in a dose-dependent manner, in the range of 0.1–10, 0.05–100, and 0.1–10 μg/mL, with a limit of detection of 0.07, 0.10 and 0.06 μg/mL, respectively. After cell incubation with mycotoxins, apoptosis occurred, which significantly affected the electrochemical signal. This approach presents a promising alternative for cytotoxic evaluation of mycotoxins.

### 2.3 Light-based bioprinting

Light-based bioprinting, uses photopolymerization to control the formation of 3D printed structures [63]. For this process to occur, a light sensitive molecule, known as photoinitiator, is necessary to produce an active species upon UV, visible, or IR light irradiation and can be manipulated to reach different depths of materials. When light strikes the photoinitiator molecule, it generates a free-radical species, which then interacts with functional monomeric or oligomeric materials in a propagative manner to produce the 3D bioprinted structure. Rapid liquid-to-solid state transition times are achieved through UV curing technology. Photopolymerization and polymer photocrosslinking occurs upon exposure to UV light and is applied during the layer-by-layer 3D printing process [64]. Notably, for biosensor fabrication, one significant advantage of light-based bioprinting is that it is a non-contact technique, eliminating the need for a nozzle, thereby reducing the risk of contamination and damage to sensitive sensor components, ensuring the integrity and reliability of biosensors. Additionally, the viscosity of the ink is not a critical factor, rendering it an exceedingly efficient and versatile bioprinting method [65,66].

Despite these advantages, photosensitive materials have shown low light selectivity (ability of the material to precisely respond to light) in light-based bioprinting. *Digital light projection* (DLP) uses a projector to project light onto a photopolymerized material for curing the layer rather than focusing on a point [67]. DLP uses a digital micromirror device to project a 2D plane of patterned light, allowing photopolymerization of an entire 2D cross-section in a single exposure. While DLP offers higher printing speed, the printable area is reduced due to the constraints of the project area and the resolution of the light mirrors. Factors such as pixel size, material polymerization properties, and movement precision impose additional restrictions on DLP. Mizaikoff and Dinc et al. applied a polymerization-induced phase separation in combination with liquid crystal display (LCD) based 3D printing on the emulsion-free fabrication of porous molecularly imprinted polymers (MIPs) in highly structured macroscopic geometries. The porous lattice cube imprinted with 17β-estradiol (E2) was used in the extraction and enrichment of the E2 hormone. Despite presenting similar surface area, 3D-printed MIPs exhibit approximately double the binding capacity for E2 compared to the porous nonimprinted control polymers after incubation analysis [68].

Wang et al. developed a novel bionic microtissue sensor to detect aflatoxin B_1_ (AFB_1_) using DLP. Liver lobule microtissues were constructed with methylacylated hyaluronic acid hydrogel, HepG2 cells, and carbon nanotubes. These microtissues were then immobilized on a screen-printed electrode for AFB_1_ detection using differential pulse voltammetry (DPV) [69].

#### 2.3.1 Volumetric bioprinting

*Volumetric Additive Manufacturing* (VAM) or Volumetric bioprinting represents a transformative advancement within the capacities of conventional bioprinting, by enabling the direct fabrication of entire structures in a single step as opposed to the sequential layer-by-layer deposition [25]. The process involves projecting 2D light patterns to selectively solidify a photosensitive material or photopolymer resin, triggering polymerization, allowing the simultaneous creation of complex structures within seconds. This approach offers advantages in terms of speed, resolution, viability, and complexity as the single-step non-contact process eliminates the necessity for multiple print passes or the use of printing nozzles, greatly reducing mechanical stress and contamination. Currently, there are relatively few photoresins available as they must be photocrosslinkable, biocompatible, optically clear and transparent in order to be used in VAM. Some resins that exhibit such properties include acrylate polymers [25], gelatin methacryoyl [70], gelatin-norbornene [71], silk fibroin and sericin [72], and silica glass [73]. The techniques' ability to produce intricate and complex structures makes it a promising technology for biomedical applications and soft tissue engineering [74]. However, compared to these applications, the use of volumetric bioprinting for biosensors development is still in the early stages of exploration and has not yet been extensively studied or implemented. Yet, the unique capabilities of volumetric bioprinting offer favourable opportunities for biosensor development, as it allows for the creation of biomimetic microenvironments that closely mimic physiological conditions, enhancing sensor sensitivity and specificity, as well as rapid prototyping capabilities of high precision, crucial for achieving optimal sensor performance. Furthermore, the integration of multiple components including sensing elements and conductive structures broadens the potential for biosensor fabrication, as demonstrated by Wolstrup et al. [75]. A novel biofabrication approach was employed by combining embedded 3D printing and volumetric additive manufacturing to fabricate conductive bioprinted structures. It involved suspending electrically conductive carbon grease within a of a mixture of two acrylate polymer components, bisphenol A glycerolate diacrylate (BPAGDA) and polyethylene glycol diacrylate (PEGDA), followed by VAM printing and post-processing which involves mechanically exposing the solid conductive carbon wire contact points. The resulting three-dimensional conductive structure exhibited a resistance of 4.5 kΩ, confirming that the conductive carbon grease remained unaffected by the resin. Further advancements to this work in the biosensor development context could include incorporating a flexible support matrix, enabling the possibility for wearable stress and strain sensors. Using polyethylene glycol diacrylate (PEGDA) and lithium phenyl-2,4,6-trimethylbenzoylphosphinate (LAP) as the photoresin, Rodríguez-Pombo et al. fabricated a drug-loaded 3D printed tablets, named Printlets™, using VAM within 17 s [76]. It was found that the drug release rates of the paracetamol-loaded Printlets could be tuned by adjusting the monomer-to-diluent ratio of the resin, whereby at lower ratio's, the drug is released more rapidly.

The rapid fabrication process, material flexibility, precision control and potential for AI integration can contribute to the development of advanced, adaptive biosensors capable of real-time monitoring and personalized diagnostics. This integration enhances the ability to tailor medical treatments and responses to individual needs, significantly improving the field of personalized medicine.

#### 2.3.2 Laser-assisted bioprinting

*Laser-assisted bioprinting* (LAB), a technique based on LIFT (Laser-induced Forward Transfer) [77], operates through three main components: the ribbon (donor slide), a laser pulse, and a receiver-slide. The ribbon comprises a layer of transparent glass, a thin layer of metal, and a layer of bioink, working together to deposit the bioink in the desired place. As the metal layer beneath the bioink vaporizes due to a laser pulse, it undergoes a phase change, facilitating the transfer of bioink from the donor-slide onto the receiver-slide. This method achieves high resolution due to the use of the laser and picolitre-sized droplets and is particularly relevant to investigating cell-cell and cell-microenvironment interactions *in vitro* since spatial organization of cells impacts their behaviour. The high precision of this technique has even been utilized to print individual cells [78]. Despite having a similar mechanism to inkjet printing, LAB, similarly to other light-based techniques, does not require a nozzle in the bioprinting process, dramatically reducing the shear stress on the cells, which yields higher rates of cell viability (>95 %) [79]. The nozzle-free operation eliminates clogging-related issues, even when using highly viscous bioinks. Even though LAB is highly promising, it is also one of the most expensive and complex bioprinting processes. To assess the impact of LAB, Karakaidos et al., carried out a comparative study on DNA damage, highlighting the effects at the sub-cellular level this bioprinting process may have on cells [80]. They found that LIFT safely printed breast cancer cells patterns with high viability with minimal heat or shear damage to the cells, indicated by unperturbed growth and negligible gross DNA damage.

#### 2.3.3 Stereolithography

Stereolithography (SLA) uses ultraviolet (UV) or visible light to cure photosensitive polymers in a layer-by-layer fashion from a vat of resin. It exhibits rapid and precise fabrication with resolutions ranging between 5 and 300 μm [81]. The resolution in the X-and Y-dimensions relies solely on the light's projection, whereas in the Z-dimension it is dictated by how rapidly the resin attenuates the light intensity through the uncured prepolymer [82]. SLA occurs when a single beam of ultraviolet light is directed by galvanometers through a transparent window into the bottom of the vat of resin, where the material is then selectively polymerized. Once a layer is finished, the printing platform is moved to allow a fresh layer of resin to flow, whereby the process starts over again until the structure is fabricated. However, the laser can also be directed as a two-dimensional shape with a digital micromirror device, in a process termed *digital light processing* (DLP). Printing time is significantly reduced with this method, as an entire layer is fabricated by turning mirrors on and off in a single exposure phase. As a result, one disadvantage of SLA is its reliance on rapid polymerization, meaning it has limited multi-material functionality [83]. Stereolithography is a rapid and highly accurate technique; but may be complex to optimize since factors such as material concentration, light type and intensity, and photo absorber sensitivities need to be considered to achieve the ideal stereolithographic system.

**Fig. 5:**
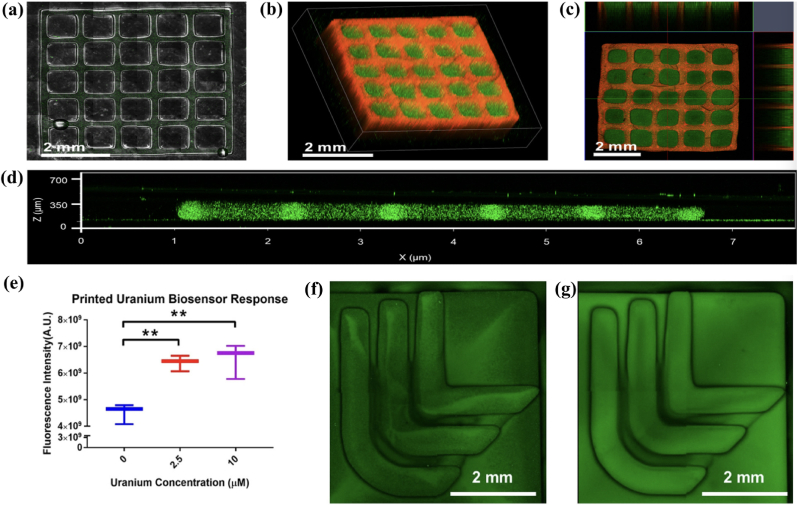
Demonstration of the engineering complexity in SLAM-printed biofilms (a) 5 by 5 unit square grid features a unit cell of 800 μm voids with 200 μm thick walls and a 2.5 mm height; (b–c) homogeneous distribution of the encapsulated fluorescent *E. coli* (green) throughout the printed construct; (d) confocal z-stacks of dual species print with encapsulated *E. coli* expressing either GFP (green) or mCherry (red); (e) fluorescence intensity for 2.5 and 10 μM uranium compared to the no uranium exposure control (f) an LLNL logo print with encapsulated C. cresentus either not exposed to uranium or (g) exposed to 10 mM uranium. The figure was adapted and reprinted from Ref. [84]. Licensee ACS (CC-BY-NC-ND 4.0). (For interpretation of the references to color in this figure legend, the reader is referred to the Web version of this article.)

Dubbin et al. presented a novel bioprinting technique to pattern microbial constructs termed ‘Stereolithographic Apparatus for Microbial Constructs’ or SLAM bioprinting [84] (Fig. 5). The SLA technique reported enabled the rapid engineering of biofilms with areas of >48 mm^2^. The printed biofilm, constructed of live microbes, demonstrated activity up to 3 days in culture, where mechanical properties such as diffusivity, substrate modulus and rheology were also investigated. Finally, to highlight the SLAM technique, the authors printed a sensor containing a modified bacterial strain, *C. crescentus* with a transcriptional fusion between the uranium-responsive promoter *Pphyt* and *gf p*. This strain fluoresces in the presence of uranium exposure and concentrations as low as 2.5 μM can be detected. This study details an interesting concept of innovating existing bioprinting technologies and demonstrates future applications in biosensor development whereby the unique capabilities of microbes can be manipulated in a controlled manner.

Bhaiyya et al. used SLA to 3D-print an electrochemiluminescence platform for the detection of H_2_O_2_ and cholesterol [85]. In only 30 min, the stereolithographic 3D printed electrochemiluminescence (GP-SE-ECL) biosensor was fabricated that exhibits a detection limit of 15.71 μM for cholesterol. A notable feature of the biosensor is its independence from an external power supply, increasing its versatility. An interference study, stability analysis and real-time sample analysis were conducted to further demonstrate the potential for practical applications. These results suggest that SLA printed devices hold great promise for biomolecule detection.

A low-cost microfluidic sensor capable of detecting and quantifying proteins from cell lysate was designed and fabricated using SLA by Sharafeldin et al. in 2021 [86]. The purpose of the printed microfluidic immunosensor is to monitor cancer metastasis protein biomarkers from a single cell. The targeted protein for detection and monitoring was desmoglein 3 (DSG3) as a metastatic marker, with vascular endothelial growth factor (VEGF) C and D serving as positive controls, and β-tubulin (β-tub) used as a loading control for evaluating the cell number in the sample. Upon recognition in the detection chamber, a chitosan-based hydrogel in the presence of cell lysate, forming a 3D structure coated with immobilized antibodies. The interaction between the antibodies and the biomarkers produced a detectable chemiluminescent signal, with results indicating that the sensor was highly sensitive for the detection of proteins, achieving a limit of detection of 0.10 fg mL^−1^ for DSG3 and 0.20 fg mL^−1^ for VEGF C, D, and β-tub. This is one of the first automated 3D-printed microfluidic immunoarray devices capable of lysing and quantifying released biomarker proteins bound to cells. It offers advantages over other single cell approaches such as its low cost, speed, accuracy, and sensitivity.

Table 1 list the most common biofabrication methods and the requirements on the development of biosensor platforms.

**Table 1:** Biofabrication techniques and important requirements for biosensors development.

BIOFABRICATION METHOD	REQUIREMENTS
Inkjet-Based	• Precise Droplet Control: Necessary for adjusting bioink formulations to best suit DOD or CIJ for accurate droplet size and placement, ensuring high resolution and reproducibility of biosensors. Strategies include exploring the use of high-quality nozzles, advanced actuation mechanisms, and tuning printing parameters. • Sterility: Implement protocols to maintain sterility during printing to reduce contamination risk for biosensors. • Even Cell distribution: Incorporate mixing mechanisms and optimize bioink viscosity to prevent cell settling and achieve uniform cell density in printed biosensor constructs.
Laser-Assisted	• Laser Parameters: Fine-tune laser parameters to avoid thermal damage to biosensor components. • Laser Absorption: Control or monitor energy absorption by the bioink or substrate to prevent unwanted reactions or degradation of biosensor materials. • Contamination Prevention: Carefully select materials and implement thorough cleaning protocols to enhance biosensor functionality when using metallic layers that absorb laser light. • Laser Focus and Alignment: Maintain laser accuracy for precise deposition of biosensor materials, critical for functionality of microscale biosensor functionality.
Stereolithography	• Sterility Maintenance: Ensure thorough post-printing cleaning protocols to remove residual photoinitiators, minimizing interference with biosensor readings. • Biocompatible Resins: Research and develop photopolymerizable materials that are biocompatible and biodegradable for safe integration within biological sensing elements. • Optical Precision: Control and calibrate the light source for accurate curing, ensuring uniform polymerization and avoiding overexposure or underexposure of biosensor components.
Extrusion-Based	• Extrusion Pressure and Temperature Control: Precisely regulate to avoid damaging sensitive biosensor components like enzymes or antibodies. • Effective Curing Processes: Implement effective crosslinking or curing methods post-extrusion to ensure timely solidification of the bioink to stabilize the biosensor construct. • Resolution Control: Ensuring printed layers are mechanically stable and properly aligned to maintain the integrity of microscale biosensor features.
Digital Light Processing	• Light Source Calibration: Regularly calibrate and control the light source to achieve consistent curing. • Production Volume: Optimize printing strategies or introduce parallel printing to maximize the number of biosensors that can be printed in a single batch, improving efficiency. • Photoinitiator Selection: Use photoinitiators that are effective for curing but do not interfere with the biological sensing components to prevent toxicity that may degrade biosensor performance. • Post-processing protocols: Implement effective steps to remove unreacted resin post-processing to prevent residual uncured resin from affecting biosensor performance.
Volumetric Bioprinting	• Complex Geometry capability: Utilize the ability to create complex structures to enhance biosensor sensitivity and functionality, which is otherwise difficult to achieve with layer-by-layer methods. • Uniform light distribution: By considering the light source, exposure time and layer thickness to ensure that the light penetrates uniformly throughout the bioink leading to consistent polymerization and reproducible biosensor constructs. • Bioink Transparency: Conductive nanomaterials used in bioinks for biosensor development are essential, but striking a balance between these often-opaque materials and maintaining the necessary transparency for light-based curing must be significantly considered when designing the bioink.

## 3 Advanced bioinks

A prominent strategy is to optimize the core material of the bioink since it dominates the functionality of the incorporated biomolecules [20]. For example, the core materials can mimic the microenvironment of the biomolecules thus maximizing their ability to bind target analytes in biosensors or preserve the protein expression profiles of entrapped cells [87].

Biocompatibility is a critical factor in bioinks, as non-biocompatible materials may trigger an immune response, leading to inflammation and consequently deactivation and denaturation of the biomolecules in cell-based bioinks or wearable sensors. In addition, bioinks can be designed to undergo triggered biodegradation through the action of incorporated enzymes (e.g., natural polymers like collagen, gelatin, silk, fibroin, among others) [88], hydrolysis (e.g., synthetic polymers such as polyesters) [89], and ion exchange (e.g., alginate and carrageenans) [90]. Triggered biodegradation facilitates temporal control of tissue development, enables tuneable degradation rates, and minimizes residual material.

These novel bioink formulations are being adapted in the context of biosensors to create functional and responsive constructs. A novel electrically conductive 3D-bioprinted biosensor was developed by Jiang et al. [91] for the sensitive detection of fish parvalbumin. The bioink was composed of polydopamine-modified multi-wall carbon nanotubes (PDA-MWCNT), gelatin methacryloyl (GelMA), mast cells and endothelial cells. This advanced bioink facilitated high-throughput 3D bioprinting of vascular microtissues, which were immobilized on modified electrodes to construct the biosensor platform. By employing differential pulse voltammetry different concentrations of fish parvalbumin were analyse and a linear detection range of 0.1 ∼ 2.5 μg/mL and a limit of detection of 0.065 μg/mL was found. The research highlights the bioinks advanced nature through its combination materials and biological components, providing enhanced conductivity, biocompatibility and a realistic simulation of human vascular structures [91]. This progression showcases the potential of advanced bioinks in developing sensitive and specific biosensors for rapid allergen detection, contributing to food safety and biomedical applications.

### 3.1 Multicomponent bioinks

A multicomponent bioink is defined as a blend of two or more biomaterials, each contributing a specific function to enhance the overall properties of the bioink. While optimizing the printability of single-component bioinks by increasing polymer concentration and crosslink density is common, these modifications can significantly compromise biocompatibility and affect transport through the printed material. Conversely, selecting a bioink based exclusively on biocompatibility may result in a viscosity that is challenging to print, leading to reduced biomolecule loading, interaction, and storage stability.

Many strategies are being adopted to improve the fabrication of multicomponent bioinks. For example, an interpenetrating network (IPN) was formed by mixing supramolecular functionalized hyaluronic acid (HA) with a covalently crosslinked methacrylated HA (Me-HA) [92]. Composite bioinks can also include weakly interacting materials. Chen et al. reported incorporating silk sericin hydrogel into an IPN with GelMA for wound dressing applications [93].

Furthermore, nanocomposite materials, including nanosilicates or supramolecular polymers, can be integrated to multicomponent bioinks to improve their mechanical and the flow properties. For instance, Chimene et al. reported a nanocomposite-reinforced bioink combining GelMA with nanosilicate to create a nanoengineered-ICE bioink with high printability and excellent mechanical properties due to its reinforced nature [94].

In a recent study, Tong et al. developed a biosensor for the electrochemical detection of H_2_O_2_ using a multicomponent bioink composed of Au nanoparticles, polydopamine, polyacrylic acid and graphene [95]. This hybrid material demonstrated excellent hydrophilicity, biocompatibility and high biomolecule loading capacity, making it an ideal platform for enzyme immobilization. A mechanically robust, stable, reproducible, and highly sensitive enzymatic biosensor was developed for both in vivo and *in vitro* determination of H_2_O_2_, with limit of detection of 0.02 μM and a wide linear range of 0.1 μM–20 mM.

Overall, multicomponent bioinks offer an innovative approach for improving the printability of bioinks without sacrificing biocompatibility or biofunctionality. However, unexpected chemical interactions in multicomponent bioink formulations can lead to the formation of harmful by-products, which may significantly compromise biocompatibility and biofunctionality.

### 3.2 Stimuli-responsive bioinks

Stimuli-responsive bioinks are a class of advanced smart materials capable of undergoing controlled physicochemical changes in response to various stimuli, including chemical (e.g., pH, nitric oxide, glucose, and redox potential), physical (e.g., light irradiation, thermal treatment, mechanical stress, electric potential, magnetic field, and water/humidity), or biological (e.g., enzymes, metabolites, and cell traction force) [96]. These changes can result in physical/chemical crosslinking, bond cleavage, and alterations in surface charge, volume, and morphology, thereby defining the physicochemical and biological features of printed bioinks [97].

Stimuli-responsive bioinks can be comprised of synthetic polymers, naturally derived components and composite biomaterials [98]. Due to their well-defined chemical structures, molecular weights, and hydrophilicity, synthetic polymers can enable more precise control of their physicochemical properties. For instance, polyethylene glycol (PEG), a synthetic polymer, is highly utilized in biomedicine due to its biocompatibility, non-immunogenicity, and resistance to protein absorption [99]. PEG can also be functionalized with thiol, methacrylate, and acrylate groups to exhibit responsiveness to UV light, thereby facilitating precise spatial and temporal control over the crosslinking process.

The sol-gel transition of silk fibroin from silkworms is an example of stimuli-responsive natural polymers. The sol-gel transition of silk fibroin, a natural polymer derived from silkworms, occurs from a combination of inter- and intramolecular interactions. This transition can occur in an aqueous solution in response to different stimuli, such as shear force, highly concentrated alcohol solution, high temperature, low pH, electric fields, and sonication. Biomolecules and nanomaterials can also be incorporated to generate sol-gel transitions [100], or to obtain a temperature-responsive bioink [101].

The development of stimuli-responsive bioinks represents a significant enhancement in the biofabrication of biosensors. Wang et al. [102] developed gelatin-based biomaterial ink with a dynamic covalent imine/Diels-Alder network and augmented with a hyperbranched triethoxysilane crossliker (HPASi), resulting in enhanced self-healing capabilities and temperature-responsive shape memory effects. Notably, they demonstrate exceptional mechanical strength, with elongation at break reaching up to 523 %, ensuring robustness during sensor fabrication. Moreover, their cytocompatibility, validated through MTT assays and laser confocal scanning microscopy, highlights their suitability for interfacing with biological systems without the adverse effects [102].

Stimuli-responsive bioinks represent a significant advancement for biosensor fabrication and technology, allowing for tailored responsiveness to environmental stimuli like pH, temperature, and biochemical signals. This integration not only expands the functional versatility of biosensors but also holds promise for the realization of highly sensitive and specific detection platforms for diverse biosensing applications.

### 3.3 Conductive bioinks

Over the past decade, conductive bioinks have emerged as leading materials for creating smart, electrically conductive bioprinted structures in tissue engineering, bioelectronics, drug delivery, and wearable sensing [103]. Ionic conductive dopants are frequently used to enhance the electronic conductivity of conductive polymers by incorporating charge carriers into the polymer networks, disrupting the stable crystal lattice backbone, and facilitating charge movement along the polymer chain.

Conductive bioinks are generally composed of conventional insulating polymer matrices for structural support, combined with conductive polymers, filler materials (such as carbon-based materials) or nanomaterials (including metal nanoparticles) that confer electrical conductivity [104]. A common strategy is to control the loading of the conducting components so that it is close to the percolation threshold. Binding of the target analyte can induce swelling or solvent ingress due to changes in the hydrophilicity/charge density, altering the separation of the conducting components and thus significantly changing the conductivity, resistivity, or capacitance of the sensor.

Poly(3,4-ethylenedioxythiophene) (PEDOT), polyaniline (PANI), and polypyrrole (PPy) are commonly used conductive polymers [105]. However, their use in tissue engineering applications is hindered by poor processability and mechanical brittleness. To overcome these limitations, several conductive polymer-based hybrid bioinks have been developed [69,91,106]. For example, polypyrrole exhibits desirable properties such as long-term stability, biocompatibility, and tuneable conductivity, making it a potential candidate for tissue engineering. However, its rigid, insoluble, and non-biodegradable nature restricts its use [107]. Combining PPy with biocompatible and biodegradable polymers, such as poly (acrylic acid), chitosan, poly (lactic acid), and alginate, can lead to blends and composites with properties suitable for bioelectronic bioinks.

**Fig. 6:**
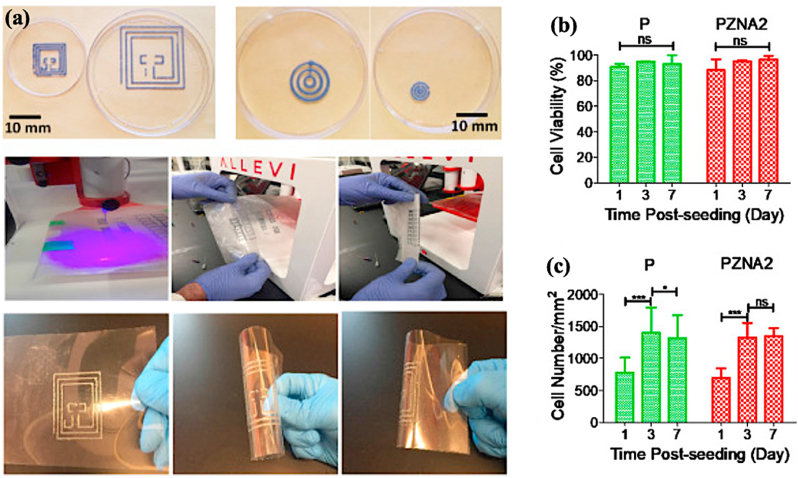
(a) 3D printing of PGSA (40 %) + sintered Zn with Acetic Acid (60 %) in different scales, reproducibility tests of the printing, and flexibility of 3D printed circuit. (b) Quantification of cell viability at days 1, 3, and 7 post-seeding. (c) Quantification of metabolic activity, and relative fluorescence units (RFU), using PrestoBlue assay at days 1, 3, and 7 post-seeding. Reprinted from Ref. [108] with permission of Elsevier.

Alternatively, incorporating electrically conductive fillers or nanomaterials, e.g., carbon-based materials or transition metal carbides/nitrides, can create highly efficient electron transport channels within the polymer matrix. These materials form covalent or non-covalent interactions with polymer chains, resulting in high conductivity. Farizhandi et al. reported a 3D printable bioink based on poly(glycerol-*co*-sebacate) (PGS) and zinc particles with an electrical conductivity of 0.0118 S m^−1^ (Fig. 6) [108]. This formulation has the potential to revolutionize the manufacture process of POC devices by enabling the development of flexible electronics and could be further adapted for use in wearable biosensor platform technologies.

Furthermore, Wang et al. [69] introduced a novel 3D bio-printed ‘liver lobule’ microtissue biosensor for the rapid detection of aflatoxin B1 (AFB_1_), a type of mycotoxin. The biosensor integrates methylacylated hyaluronic acid (HAMA) hydrogel with HepG2 cells and introduces multi wall carbon nanotubes to improve the conductivity. The liver lobule microtissue was 3D-bioprinted and immobilized onto a screen-printed electrode, enabling the electrochemical detection of AFB_1_ using differential pulse voltammetry. The sensor exhibited a linear detection range of 0.1–1.5 μg/mL and a calculated lowest limit of detection of 0.039 μg/mL. By combining materials that complement each other in terms of biocompatibility and conductivity, this approach not only advances mycotoxin detection but also demonstrates the potential of using conductive bioinks in 3D bioprinting for biosensor development.

## 4 Bioinks for biosensors

**Fig. 7:**
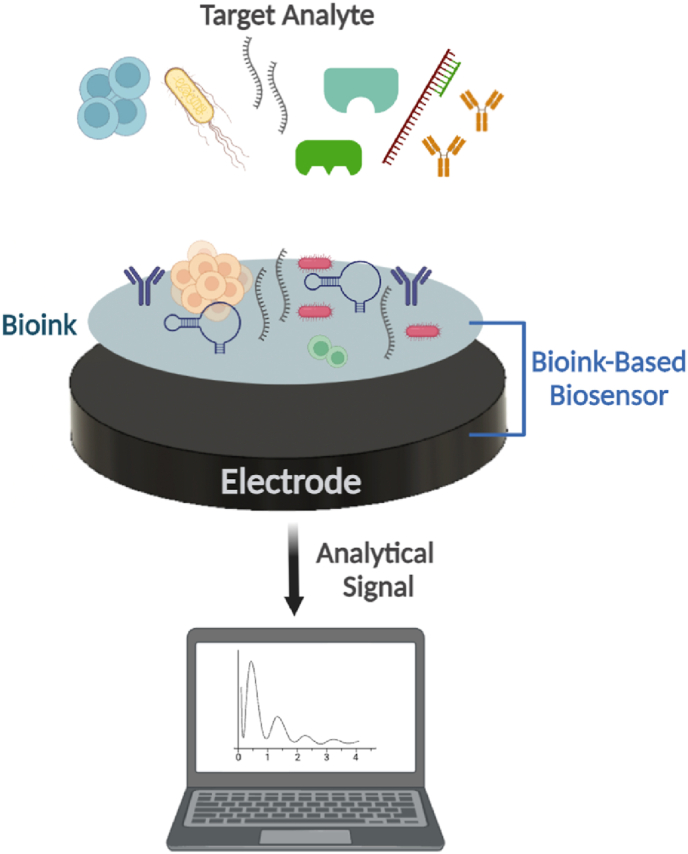
Schematic representation of a bioink-based biosensor and its key element. Created with BioRender.com.

Biosensors are integrated receptor-transducer platforms that can convert biological or chemical reactions into optical or electrical signals proportional to the analyte concentration. They consist of three essential parts: (1) the bioreceptor (e.g. antibody, enzymes, nucleic acids, cells or microorganisms); (2) the transducer of the physicochemical signal (e.g. electrochemical, mass, thermal, or optical signal), and (3) a signal processor to interpret the converted information [109] (Fig. 7). To obtain reliable, reproducible and accurate analytical signals, the bioreceptor must remain bioactive and stable after immobilization via physical adsorption, covalent bonding, crosslinking, or encapsulation [110,111]. Therefore, bioprinting technologies have emerged as powerful biofabrication platforms for the development of novel bioink-based biosensors. This fabrication methodology can improve the stability and reproducibility of complex bioprinted constructs.

### 4.1 Bioink-based biosensors - an overview

Bioink-based biosensors are analytical platforms where a biomaterials ink containing bioactive bioreceptors, such as enzymes, antibodies, nucleic acids, or cells, is deposited onto an electrically conductive substrate using biofabrication techniques. Preserving the bioactivity of the sensing element is fundamentally important, and the bioreceptor must remain bioactive, stable, and, most importantly, available to bind the target analyte.

Among the most historically significant biorecognition elements are *enzymes*. For instance, the success of the glucometer, representing about 90 % of the global biosensor market, relies on enzymes like glucose oxidase or glucose dehydrogenase [112]. A plethora of different target analytes can be detected by selecting the appropriate enzyme (e.g.*,* oxidoreductases for lactate, malate for cholesterol quantification; transferase for xenobiotic determination or ligase for DNA mutation identification) and combining them with an electron transfer mediator within a conducting matrix. The simplest detection mechanism is the selective electrocatalytic oxidation or reduction of the target analyte, but enzymes are also very useful as labels in antibody and nucleic acid sandwich type assays.

Additionally, *antibodies* can determine the target analyte, *i.e.,* antigen, through highly specific binding. The introduction of ELISA (enzyme-linked solid phase immunoassay) enabled the monitoring of cancer cells [113] and other important biomarkers [114] using a direct or indirect detection strategy [115]. The strong base pair affinity between complementary nucleotide strands is a detection mechanism widely exploited in biosensor development. Oligonucleotide sequences, such as *DNA, RNA,* or *peptide nucleic acid,* represent another important class of sensing elements. Particularly noteworthy is the introduction of *aptamers*, oligonucleotide sequences that revolutionized biosensor technology. In fact, by using synthetic techniques (*i.e.* SELEX, systematic evolution of ligands by exponential enrichment), it is possible to obtain artificial single-stranded DNA or RNA oligonucleotides that specifically bind targets such as proteins, bacteria, small molecules, in contrast to the first generation of DNA-based biosensors used only as genosensors to identify a pair match/mismatch for genetic disorders.

**Fig. 8:**
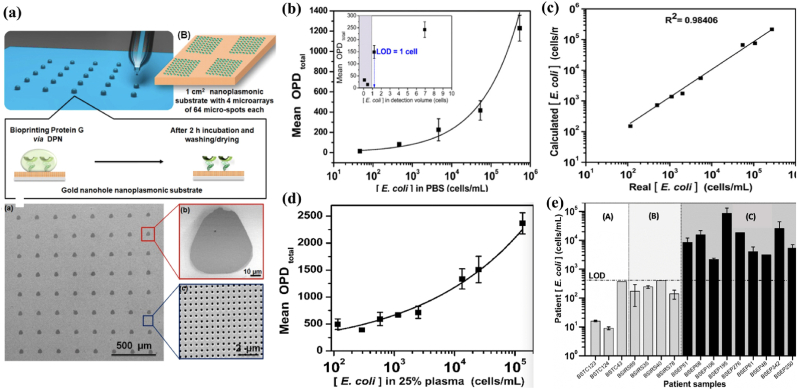
**(a)** Schematic illustration of the bioprinted process using dip-pen nanolithography-based spotter onto the nanoplasmonic substrate; (b) Calibration curve of *E. coli* in PBS without preconcentration (i.e., without bacteria enrichment) in a wide dynamic range of 10–10^6^ cells/mL. Inset: Plot indicating the experimental LOD in terms of cells in a constant detection volume of 150 μL; (c) Correlation plot of real and calculated concentrations of blind spiked bacterial samples in PBS; (d) Calibration curve in 25 % (v/v) diluted plasma in PBST. All Y-axes represent mean OPD_total_ values; (e) Clinical evaluation of the diagnostic assay with real patient samples. Measurements were done in situ at the hospital settings: (A) healthy controls, (B) SIRS patients serving as controls for non-infectious disease, and (C) sepsis patients. Reprinted (adapted) with permission from Ref. [116]. Copyright 2024 American Chemical Society.

Moreover, *cells* or *microorganisms* adopted as sensing elements enable numerous applications, particularly in the detection of pathogenic or cytotoxic species, performing dynamic, rapid, and real-time bioassays. For example, Estevez et al. developed a bioink-based PoC biosensor for E. Coli quantification (Fig. 8) [116]. A highly viscous glycerol matrix containing protein G was directly bioprinted using dip-pen nanolithography-based spotter onto the nanoplasmonic substrate. This microarray-based biosensor allowed the direct label-free quantification of *E. coli* by exploiting the lens-free interferometric microscopy. The *E. coli* concentration was sensitively determined in buffer (i.e., without bacteria enrichment) in a wide dynamic range of 10–10^6^ cells/mL and diluted plasma with only one-step sample handling. Analysis performed in 10 μL volume of sample presented a limit of detection (LOD) of 100 cells/mL, while using a volume of 150 μL generated a LOD of 8 cells/mL. This technology shows good capabilities for discriminating sepsis patients (when *E. coli* was the causative agent) from healthy controls in clinical trials. However, due to the optical properties of the patient plasma itself, the sensor suffers from the limitation of signal variability, which can show high signals from control groups (healthy patients) increasing the possibility of a false positive responses. Despite some limitations, this study demonstrates that advanced bioinks can pave the way for the implementation of modern PoC devices, thereby improving on-site testing of pathogens for clinical diagnosis.

**Fig. 9:**
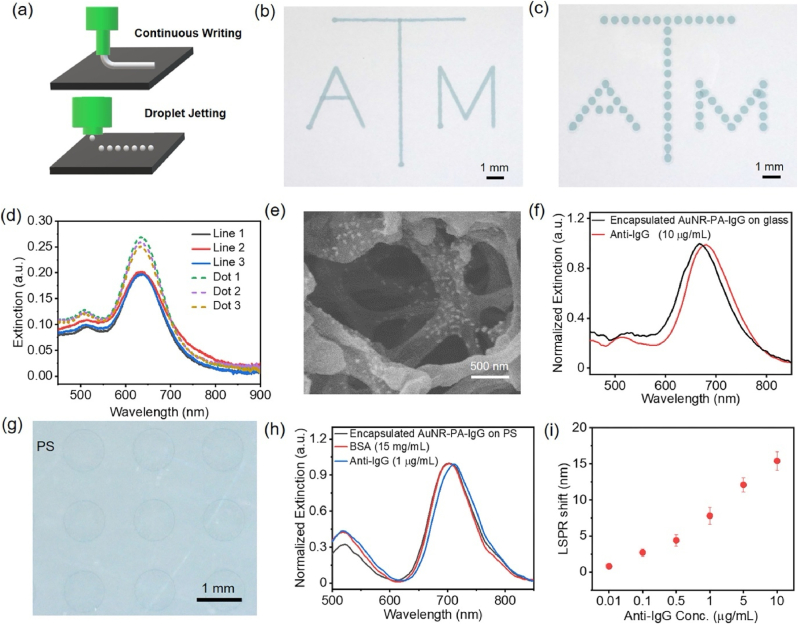
Printing the plasmonic bioink with direct writing techniques. (a) Schematic illustration showing two different direct writing techniques, including continuous writing and droplet jetting. The logo of Texas A&M University printed with the AuNR ink on a nitrocellulose membrane via (b) continuous writing and (c) droplet printing. (d) Extinction spectra collected at different positions of (b) lines and (c) dots in the logo showing the excellent spectral uniformity of the printed patterns. (e) SEM image showing the uniform distribution of AuNR on the nitrocellulose membrane. (i) Extinction spectra of the encapsulated AuNR–PA–IgG bioink printed on a glass substrate before and after exposure to anti-IgG of 10 μg/mL. (g) Optical image of the printed dot array of encapsulated AuNR–PA–IgG on a polystyrene (PS) plate. (h) Extinction spectra of the encapsulated AuNR–PA–IgG printed on the PS plate before and after exposure to anti-IgG of 1 μg/mL and after exposure to BSA of 15 mg/mL (i) LSPR shift of the encapsulated AuNR–PA–IgG printed on the PS plate after exposure to anti-IgG of varying concentrations. Error bars represent the standard deviations from three replicates. Reprinted (adapted) with permission from Ref. [117]. Copyright 2024 American Chemical Society.

Tian and collaborators [117] developed a thermically ultrastable plasmonic bioink by encapsulating antibodies in an organosiloxane polymer through in situ polymerization (Fig. 9). It exhibited excellent thermal, biological, and colloidal properties with superior stability compared to biochips with unencapsulated antibodies. The biomanufacturer process applied to obtain the plasmonic biochips goes beyond bioprinting technologies, demonstrating that bioink-based biosensors can also be reliably manufactured by in situ polymerization of a highly stable PoC biosensor device capable of sensitivity and selectivity quantifying a protein biomarker at clinically relevant concentrations.

**Fig. 10:**
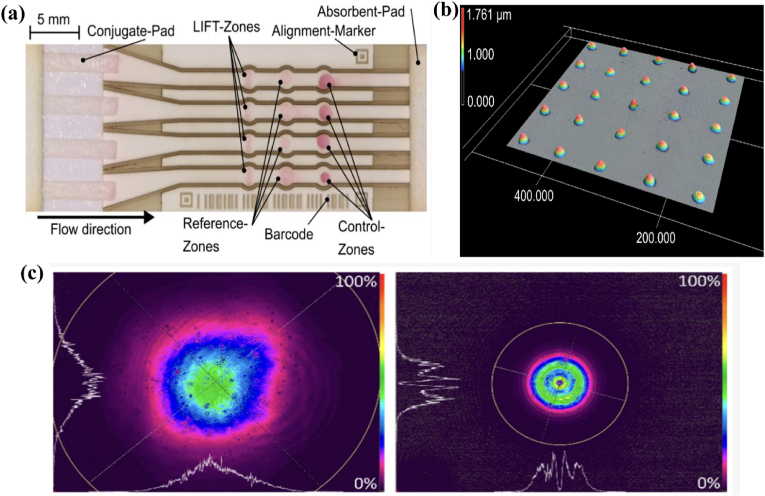
(a) Prototype of fully laser fabricated multichannel lateral flow test. The laser structured nitrocellulose membrane consists of 4 parallel channels with 3 reaction zones each. For the proof of concept with CRP detection the first zone of each channel is functionalized with 120 nL of capture antibody solution (24,000 droplets with an individual volume of 5 pL) by BA-LIFT, while the other zones, which appear far less precisely defined, are manually spotted; (b) 3D-LSM image of a 5 × 5 array of ink droplets transferred by BA-LIFT onto a glass substrate; (c) Beam profiler images (recorded with Ophir Spiricon SP928 camera and BeamGage software) of the laser pulses used for the blister-actuated laser-induced forward transfer (BA LIFT) process measured before the focusing optics. The figure was adapted and reprinted from Ref. [118]. Licensee MDPI, Basel, Switzerland (CC-BY 4.0).

Similarly, a multichannel lateral flow colorimetric device capable of detecting C-reactive protein (CRP) was fabricated by Hecth and Dietzel et al. (Fig. 10) [118]. A polyimide bioink was deposited on the nitrocellulose surface using blister-actuated laser-induced forward transfer (BA-LIFT). The first zone of each channel of laser-structured nitrocellulose membrane was functionalized with a capture antibody solution. Changes in colour were observed when CRP was identified. Despite the use of an intense laser source, the printed materials and receiver substrates were not degraded by optical or thermal effects.

Another notable development is the fully printed biosensor with tyrosinase-containing bioink for detecting the pollutant catechol in natural water samples, developed by Cagnani and collaborators [119]. The biocompatible bioink was prepared by dispersing the carbon black and the carboxymethylcellulose in phosphate buffer saline. After, tyrosinase enzyme was incorporated into the mixture. Two high through-put manufacturing coating techniques were combined, termed slot-die printing and roll to roll printing, in order to deposit the bioink onto the working electrode surface of screen-printed carbon electrodes (SPCEs). Whereby, slot-die deposition enables enzymes to be printed without significant activity loss and roll-to-roll allows for controllable adjustment of the ink thickness. Following the deposition, electrochemical measurements were performed to verify the tyrosinase catalytic activity. It was demonstrated that enzymatic activity was not altered by the bioprinting process. Additionally, the bioink-modified electrodes were used as sensors to detect dopamine, serving as proof-of-concept for the applicability of the proposed approach. The methodology illustrated by Cagnani opens new prospects for large-scale production of disposable devices due to the versatility of the deposition/printing approach.

The advantages of using aptamers in the bioink formulation were clearly illustrated by Stanciu and Diaz Amaya [48]. A carboxyl-functionalized aptameric bioink on a nitrocellulose substrate for the highly efficient detection of *E. coli* via a sandwich format was developed. Changes in conformation led to a colour response on the nitrocellulose strip that was visualized when the aptamer recognizes *E. coli*.

Issues such as thermal denaturation or mechanical damage can be easily overcome by changing the biofabrication process, *i.e.* using a piezoelectric inkjet printer, or by improving the bioink crosslinking strategy. Additionally, the biomolecules in the printed top layer have a short shelf-life (denaturation or loss of bioactivity) due to the environment exposure, e.g.*,* temperature, oxygen and reactive species, pH, light, and moisture exposure. To improve the lifetime of the biolayer, Weng and collaborators [120] entrapped the horseradish (HRP) enzyme between two layers of polypyrrole (PPy) and ethyl cellulose (EC). The amperometric biosensor was applied to detect hydrogen peroxide and glucose.

A comprehensive list of the most recent papers in the context of bioink for biosensors is illustrated in the table below (Table 2).

**Table 2:** List of the most recent bioink-based biosensors widespread in literature.

Bioink composition	Printing technique	Bioreceptor	Type of biosensor	Target	LOD	Ref.
PVAm + Enzyme	Inkjet printing	AChE	Lateral flow colorimetric	paraoxon, aflatoxin B1	paraoxon ∼100 nM; aflatoxin B1,∼30 nM	[121]
Glycerol + Protein	Nanolithography	Protein G	Nanoplasmonic	*E. Coli*	400 cells/mL	[116]
Polyimide + Monoclonal antibodies (mAb)	Laser	Human C reactive protein	Paper-based colorimetric	C reactive protein	–	[118]
PVA + Bacteria	Inkjet printing	–	Time-temperature colorimetric	*G. stearothermophilus, B. atrophaeus*	–	[122]
Carbon black and carboxymethylcellulose + Enzyme	Inkjet printing	Tyrosinase	Electrochemical	Catechol	0.09 μmol L^−1^	[119]
Organoxilane polymer + AuNR−PA−Ab	Direct writing	IgG	Plasmonic	Protein A	10 ng mL^−1^	[117]
Carboxyl-functionalized aptameric solution	Inkjet printing	Aptamer	Lateral flow colorimetric	*E. Coli*	233 CFU mL^−1^	[48]
Polypyrrole + enzymes	Inkjet printing	GOx and HRP	Electrochemical	Glucose and H_2_O_2_	1–5 nM (glucose) 10 μM–10 mM (H_2_O_2_)	[120]
Ab solution	Inkjet printing	Murine monoclonal anibodies (mAb)	Lateral flow colorimetric	Ovalbumin	<1–25 ng mL^−1^	[123]
AgNCs@Prussian blue + Enzyme	Screen-printing	Oxidase	Electrochemical	Glucose	0.005 mM	[124]
Enzyme solution	Inkjet printing	Acetylcholinesterase (AChE)	Paper-based colorimetric	Organophosphorus pesticides	–	[125]
Poly(methyl methacrylate) (PMMA) + Ab	Microcontact printing	Antibody	Immunofluorescent	Interleukine-6 (IL-6)	0.5 pg mL^−1^	[126]
Xerogel + Enzymes	Inkjet printing	GOx/peroxidase POx and Uricase/POx	Sol-gel-sol colorimetric	Glucose and uric acid	<0.02 mM for glucose	[127]
CNTs, magnetic NPs + anti-mAb	Magnetic-3D printing	mAb c-Myc	FET	Anti-c-Myc	pM range	[128]
GelMA, PEGDMA + Cells	Inkjet printing	Chondrocytes	Electrochemical	2,4,6-Trinitrotoluene (TNT)	0.38 pg mL^−1^	[129]

### 4.2 Challenges in the development of bioink-based biosensors

Despite the significant progress, a limiting factor in bioprinting is the shortage of bioinks with specific physicochemical characteristics and intrinsic bioactivity while maintaining biocompatibility and mimicking the extracellular matrix (ECM) after the printing process [130].

A fundamental challenge in the development of bioink-based biosensors is the availability of advanced functional material that can provide specific characteristics to the bioink formulation deemed crucial for the functioning of the sensor platform, e.g., conductive fillers for electrochemical biosensors, without compromising the printability, mechanical properties, biomolecule bioactivity, long-term stability and cytotoxicity. Moreover, bioinks with high crosslinking densities or stiffness can inhibit the functionality of encapsulated biological sensing components, e.g., deactivation of bioactivity by compact biomolecule encapsulation [131], as well as poor mass transport properties.

Optimizing bioink composition to maintain a balance between mechanical stability and biofunctionality introduces another layer of complexity to the formulation. For example, to improve conductivity, an essential feature for electrochemical biosensors, the conductive filler percentage must be close to the percolation threshold, which can significantly impact the viscosity, and thus printability, biolayer thickness, and charge transfer ability. Higher thickness can hinder electron transfer between the electrode and the bioreceptor due to the longer pathways, imposing a greater barrier to electrons movement, thereby slowing the overall charge transfer rate. Conversely, thinner bioink layer can enhance mass transport by reducing the diffusion distance, leading to improved sensitivity [132]. However, thin bioink layers may lack the mechanical stability required to maintain the integrity of the biosensor particularly in dynamic or harsh environments. In addition, thin bioink layers can present durability challenges over time due to physical shear stress being more susceptible to rupture, delamination or deformation, which can compromise the reliability and longevity of the biosensor. Thinner layers often do not provide sufficient support for the embedded biological recognition elements, potentially affecting their functionality and stability. Additionally, porous structures exhibit a higher permeability which enable faster response times and improved sensitivity. However, formulating bioinks with appropriate porosity requires careful consideration of the mechanical properties. In contrast, excessive porosity can weaken the bioink structure potentially leading to mechanical failure or detachment from the sensing elements. Therefore, a balance must be found to ensure that the bioink is sufficiently porous to enhance mass transport while maintaining the mechanical integrity necessary for reliable sensor performance [133].

The thickness of the bioink layer often imposes considerable mass transport barriers, blocking the target analyte diffusion towards the sensor surface, impacting the sensitivity, and increasing the sensor response time due to electrical passivation effects. To maintain the sensitivity and accuracy of biosensors, an antifouling type of material should be selected to prevent biofouling [134].

In terms of bioprinting, high resolution and printing precision are crucial factors that can directly impact the biosensor performance, e.g., reproducibility in biosensor miniaturisation. Introducing variability to the biofabrication can promote inconsistency of sensor performance across different printing runs leading to low reproducibility, reliability and accuracy of the bioprinted structures [135]. The main component that characterises a bioink is the biomolecule incorporated into the core biomaterial and it must remain bioactive after the biofabrication process. The bioprinting conditions can significantly impact the bioactivity of the incorporated biomolecules. For instance, higher temperatures (>37 °C) can denature and deactivate biomolecules and shear forces during biofabrication can lead to biomolecules damage [[136], [137], [138], [139]]. The storage and handling condition will also determine the long-term stability and remaining bioactivity of the bioink.

### 4.3 Future directions

Bioink-based biosensors represent a groundbreaking integration of materials science, biology and engineering, having the potential to significantly impact healthcare, diagnostics, environmental monitoring, animal free drug testing and personalized medicine. Bioprinting technologies can leverage the fabrication of spatial patterns with diverse (nano)materials and biomolecules, effectively mirroring the complexity of human microenvironments [140].

The combination of the advanced material with a sustainable biofabrication technique could be an important step to shorten the gap between biosensor and robust device construction. Despite the broad spectrum of printable materials, only a subset of these materials can be used for biosensing, meeting the criteria of biocompatibility, specific affinity for the target molecule, and suitability for processing flow that matches a particular viscosity range. For these reasons, the science concerning the advancement of bioinks for use in biosensor applications is undergoing dynamic evolution.

Novel advanced biocompatible materials, can accelerate the development of tailored multifunctional bioinks to enable the detection of multiple analytes, simultaneously, improving both diagnostic and therapeutic capabilities. Recently, bioreceptor-based bioinks have been used as the core material for the biofabrication of biosensor platforms in order to improve specificity and binding affinity against the target analytes [117,126,[141], [142], [143]]. In regenerative medicine, bioink-based biosensors have been applied to monitor tissue regeneration and integration and ensure that tissues and organs have been successfully implanted. To address limitations in wearable and implantable devices, 4D bioprinting, has emerged as a transformative technology enabling the development of smart platforms that can dynamically respond to changes when a stimulus is applied, e.g., temperature, humidity, pH, light, or magnetic fields [144]. However, designing and fabricating 4D bioprinted biosensors with precise and reliable functionality is complex and requires extensive investigation.

Additionally, to achieve high precision, advancements in bioprinting technologies are required to produce more detailed and accurate sensor designs at the microscale. Furthermore, a quality control model enabled by machine learning to monitor and verify the biofabrication process stability and defect detection within the printed structure could also overcome variability issues in printed biosensors [55,145]. The scalability and integration of these devices are pivotal in translating laboratory breakthroughs into tangible medical solutions, ensuring their practical application in real-world scenarios.

## 5 Conclusion

Here, an overview of bioink-based biosensors was presented. Firstly, the bioprinting methods were outlined to highlight the advantages, disadvantages and unique considerations of the currently available biofabrication techniques that can be applied for the fabrication of biosensor devices. Then, the features and properties of the most relevant advanced bioinks were presented. In addition, the use of the advanced bioinks for the fabrication of novel biosensors was highlighted, showcasing scientific progress in this relatively unexplored field. Lastly, the current challenges and future directions were assessed.

Recent progress in bioprinting techniques has facilitated the fabrication of more complex and sophisticated biosensors capable of better mimicking biological systems. Integrating advanced bioinks, such as those containing stem cells or other biologically active materials, has the potential of transforming biosensor design and enhancing diagnostic capabilities. This advancement could streamline multiplex operations and reduce reliance on costly instrumentation.

Furthermore, tailored development of novel bioinks, designed to enhance signal transduction or target specific molecules, will play a pivotal role in advancing biosensors across a wide range of applications, including drug discovery, environmental monitoring, food quality evaluation and personalized medicine. Nevertheless, further research is needed to refine these next generation bioinks and ensure their safety and effectiveness for use in clinical settings.

In essence, the progress in advanced bioinks and bioprinting techniques has the potential to significantly improve the reproducibility, functionality, and sensitivity of biosensors, thereby, holds promise for revolutionizing medical diagnosis and treatment, opening new avenues for innovation in healthcare.

## CRediT authorship contribution statement

**Róisín Byrne:** Writing – review & editing, Writing – original draft, Visualization, Methodology, Investigation, Data curation, Formal analysis. **Amanda Carrico:** Writing – review & editing, Writing – original draft, Validation. **Mariagrazia Lettieri:** Writing – original draft. **Athira K. Rajan:** Writing – original draft. **Robert J. Forster:** Writing – review & editing, Supervision, Conceptualization. **Loanda R. Cumba:** Writing – review & editing, Writing – original draft, Supervision, Resources, Project administration, Funding acquisition, Conceptualization.

## Declaration of competing interest

The authors declare the following financial interests/personal relationships which may be considered as potential competing interests: Loanda R. Cumba reports financial support was provided by 10.13039/501100001602Science Foundation Ireland. If there are other authors, they declare that they have no known competing financial interests or personal relationships that could have appeared to influence the work reported in this paper.

## Data Availability

No data was used for the research described in the article.
